# Social stress during adolescence activates long-term microglia inflammation insult in reward processing nuclei

**DOI:** 10.1371/journal.pone.0206421

**Published:** 2018-10-26

**Authors:** Marta Rodríguez-Arias, Sandra Montagud-Romero, Ana María Guardia Carrión, Carmen Ferrer-Pérez, Ana Pérez-Villalba, Eva Marco, Meritxell López Gallardo, María-Paz Viveros, José Miñarro

**Affiliations:** 1 Department of Psychobiology, Faculty of Psychology, Universitat de València, Valencia, Spain; 2 Department of physiology, Faculty of Medicine, Complutense University of Madrid, Madrid, Spain; 3 Department of Animal Physiology, Faculty of Biological Sciences, Complutense University of Madrid, Madrid, Spain; University of Texas, UNITED STATES

## Abstract

The experience of social stress during adolescence is associated with higher vulnerability to drug use. Increases in the acquisition of cocaine self-administration, in the escalation of cocaine-seeking behavior, and in the conditioned rewarding effects of cocaine have been observed in rodents exposed to repeated social defeat (RSD). In addition, prolonged or severe stress induces a proinflammatory state with microglial activation and increased cytokine production. The aim of the present work was to describe the long-term effects induced by RSD during adolescence on the neuroinflammatory response and synaptic structure by evaluating different glial and neuronal markers. In addition to an increase in the conditioned rewarding effects of cocaine, our results showed that RSD in adolescence produced inflammatory reactivity in microglia that is prolonged into adulthood, affecting astrocytes and neurons of two reward-processing areas of the brain (the prelimbic cortex, and the nucleus accumbens core). Considered as a whole these results suggest that social stress experience modulates vulnerability to suffer a loss of glia-supporting functions and neuronal functional synaptic density due to drug consumption in later life.

## Introduction

Substance abuse and addiction are complex processes in which stress plays a critical role [1, 2, 3, 4, 5]. Addiction and stress responses share a common neurobiological pathway which can be modified by environmental stressors [6, 7]. Vulnerability to relapse into drug-seeking following a stress-producing experience, even after long periods of abstinence, highlight the power of stress-induced neurobiological modifications [8]. The plasticity of the adolescent brain may be one of the main factors involved in the elevated percentage of drug use initiation and rapid development of addiction disorders in this period of life [9]. During adolescence, the prefrontal cortex and limbic regions undergo a maturation process characterized by myelination and competitive synaptic elimination [10, 11]. The immaturity of the frontal cortex circuitry revealed by neuroimaging studies partially explains why teens are more responsive to rewarding experiences and experience them positively over any negative attribution [12, 13]. The adolescent brain is characterized by a pro-motivational state; this is the result of a limited inhibitory capacity, high dopamine release in the nucleus accumbens (NAc) when processing positive stimuli, and an overactive amygdala [14].

The risk of developing psychological disorders such as depression and anxiety and increased vulnerability to drug use can be attributed to the experience of social stress during adolescence [15,16]. Exploring the mechanisms by which social stress affects health requires appropriate animal models. The rodent social defeat model has been widely employed in this sense, and numerous studies using this model have provided knowledge of the neurobiology and behavioral changes related to this type of stress [17, 18]. The procedure for inducing social defeat is based on the resident/intruder paradigm in which the intruder animal is placed in the cage of a resident/aggressive rodent that attacks and threatens it. These agonistic interactions promote the development of dominance-based social hierarchies [19]. The repeated social defeat (RSD) model is a putative model of bullying with face validity [20, 21] that induces strong physiological, behavioral and endocrine responses [22, 23, 24, 25].

Research has repeatedly shown that social defeat induces an increase in the rewarding effects of cocaine. Defeated animals show higher acquisition and maintenance of cocaine self-administration [26, 27, 28] and quicker escalation of cocaine-seeking behavior [29, 30, 31, 32]. In addition, social defeat increases conditioned place preference (CPP) induced by cocaine [33, 34, 35, 36, 37, 38]. We have previously reported that experienced social defeat during adolescence also augments the conditioned rewarding effects of cocaine in adult animals and modifies cocaine self-administration [22, 23].

There is an effective communication between the peripheral immune system and the central nervous system (CNS), and, in this context, chronic or intense stress experiences can induce a proinflammatory state [39]. In fact, stress-related psychiatric disorders are hypothesized to be due to this proinflammatory state in the CNS [40]. Human and animal studies show that immune mediators are able to influence the way the brain processes information and responds to it [41]. One example is the relationship of neuroinflammation with the pathophysiology of depression, which has been deeply studied (review on [42]). Peripheral inflammatory responses can access the brain, contributing to the increase in neurotoxic kynurenine pathway metabolites and the decrease in neuroprotective metabolites. Activated microglia released glutamate join to the kynurenine metabolites that stimulates the N-methyl- D -aspartate (NMDA) receptors. In addition, inflammatory mediators can also downregulate dopaminergic neurotransmission via oxidative stress and mitochondrial dysfunction. Activation of NMDA receptors and deficient dopaminergic neurotransmission both result in depression symptoms. Proinflammatory cytokines can also exert direct neurotoxic effects on specific brain regions [43]. Previous imaging studies have reported associations between proinflammatory states and alterations in brain regions involved in emotional regulation, including the hippocampus, the amygdala and the anterior cingulate cortex [44]. In addition to these effects, cytokines are expressed in the CNS constitutionality and serve as important plasticity factors in the formation and stabilization of neuronal circuits during development [43]. Numerous studies have shown that RSD induces microglial activation and increased cytokine production [45, 46].

Research into stress induced-neuroinflammation needs to address how long these changes endure in the brain. For example, anxiety-like behavior induced by RSD can be observed immediately after the last defeat and persists for a week, but seems to vanish 3 weeks later, temporally correlating with the neuroinflammatory response [47]. Conversely to these results, we and other authors have repeatedly shown that sensitivity to the rewarding effects of cocaine continues to be increased one month after the last social defeat [22, 23, 37, 48, 49], regardless of the age at which social defeat is experienced. Changes in the blood brain barrier (BBB) structure after an experience of RSD during adolescence are also observed one month after the last encounter. The NAc and hippocampus of defeated adult mice display reductions in the expression of claudin-5 (a tight junction protein) and a higher degradation of basal laminin (decrease in laminin and collagen-IV expression) [22]. Other studies have linked disruption of the integrity of the BBB [50, 51] to increased levels of proinflammatory cytokines [52] or free radical formation [53].

Neuronal-supporting glia like astrocytes and microglia play a critical role in maintaining the BBB. Microglia are immune cells resident in the CNS and are sensitive and reactive to disruption of homeostasis [54, 55, 56]. The resting phenotype, observed in basal/healthy conditions, is characterized by a ramified morphology with a small round soma. However, in response to harmful stimuli, microglia change their shape towards an amoeboid structure with a reduction of processes length, and short-term proliferation reactivity [57, 58, 59]. The ionized calcium binding adaptor molecule 1 (Iba1), expressed in reactive and quiescent cells, has been widely used as a protein marker of microglia [60]. Both classic inflammatory stimuli and psychological stress have been shown to induce changes in microglia [61, 62, 63, 64]. However, stress-related studies have taken measurements a short time after stress exposure, and so little is known about how microglia reactivity might be maintained in the long-term.

The correct functionality of the BBB requires astrocytes, which secrete factors that promote a tight association between the cells [65]. As both astrocytes and microglia are highly sensitive to inflammatory signals produced by stress, a strong impact could have long-lasting consequences that persist with age and consistently affect neuronal survival [66, 67, 68]. Synaptophysin is considered the most important glycolipid protein in the structure of vesicle membranes in the axon terminal [69], and it is a molecular indicator of synaptic density. Decreased levels of synaptophysin is related to loss of synaptic contacts and are associated with functional deficit, while increased levels are related to synaptic plasticity and structural changes [70].

The aim of the present work was to describe the long-term effects induced by RSD during adolescence on the neuroinflammatory response and synaptic structure by evaluating different glial and neuronal markers. We hypothesized that social stress experience during adolescence will induced a neuroinflammatory response that can account for the increased observed in the rewarding effects of cocaine. Our results confirm that, when experienced in adolescence, RSD produces inflammatory reactivity in microglia that is prolonged into adulthood, seriously affecting the astrocytes and neurons of two reward-processing areas of the brain: the PrL and the NAc.

## Material and methods

### Animals

A total number of 104 OF1 male mice (Charles River, Barcelona, Spain) were used in this study. The experimental mice (n = 84) arrived at the laboratory at 21 days of age and were housed under standard conditions in groups of four in plastic cages (27×27×14 cm) during the entire experimental procedure. Mice employed as aggressive opponents (N = 20) were housed individually in plastic cages (21 × 32 × 20 cm) for a month before the start of the experiments with the purpose of heightening their aggression [71]. The housing conditions were as follows: constant temperature; a reversed light schedule (white light on 8:00 to 20:00); and food and water accessible ad libitum, except during behavioral tests. The experimental protocol has been approved by an Institutional Review Committee for the use of animal subjects (Comité d'Ètica d'Experimentació i Benestar Animal). Procedures involving mice and their care were conducted according to national, regional and local laws and regulations, which are in compliance with the Directive 2010/63/EU. All the efforts were made to minimize animal suffering and to reduce the number of animals used.

### Drugs

For cocaine treatment 1 or 25 mg/kg of cocaine hydrochloride (Alcaliber laboratory, Madrid) were used. The low dose of cocaine was selected on the basis of previous CPP studies showing that 1 mg/kg is a threshold dose [72, 73, 74] that has rewarding effects depending on the state of the mouse’s brain reward system. The dose of 25 mg/kg is an effective rewarding dose which shows reinstatement of the extinguished preference with a priming dose of 12.5 mg/kg of cocaine [75]. All treatments were adjusted in a volume of 0.01ml/g of weight. Control groups were injected with physiological saline (NaCl 0.9%), which was also used to dissolve the drugs.

### Experimental design

Two different sets of mice were employed in this study: 54 mice underwent the CPP procedure and 30 mice received the same pharmacological treatment and were employed to obtain brain samples. The total sample of 84 experimental mice was divided into six experimental groups according to the stress condition (exploration vs social defeat) and the cocaine dose used during cocaine treatment (saline, 1 mg/kg and 25 mg/kg). Social defeat or exploration began on PND 26 to 35 and the CPP procedure (PND 53–64) or cocaine treatment (PND 60–63) was initiated three weeks after the last social defeat. Brain samples were obtained one day after the last cocaine or saline injection on PND 63. A detailed outline of the experimental procedure is provided in Table 1.

**Table 1:** Experimental design.

GROUPS	(n = )	Social defeat				3 weeks		Experimental Procedure	
		1st	2nd	3rd	4th				
PND		26	29	32	35		53-54-55	56–62	63
**EXPLORATION**	14	**Exploration without conspecific**					**Pre-C test**	CPP: 1mg/kg Cocaine	**Post-C test**
	17							CPP: 25mg/kg Cocaine	
	5							Saline	**Brain Samples**
	5							CPP: 1mg/kg Cocaine	
	5							CPP: 25mg/kg Cocaine	
**RSD**	11	**Social defeat**					**Pre-C test**	CPP: 1mg/kg Cocaine	**Post-C test**
	12							CPP: 25mg/kg Cocaine	
	5							Saline	**Brain Samples**
	5							CPP: 1mg/kg Cocaine	
	5							CPP: 25mg/kg Cocaine	

### Apparatus and procedures

#### Repeated social defeat encounters

We exposed animals in the RSD groups to 4 episodes of social defeat lasting 25 min each. The episodes consisted of 3 phases that began by placing the animal or intruder in the aggressive opponent’s or resident’s home cage for 10 min. During this initial phase, the intruder was kept safe from attack by a see-through wire mesh wall that allowed for social interactions and species-typical threats from the male aggressive resident [76]. In the second phase, the wire mesh was removed and a 5-min period of confrontation followed. In the third phase, the wire mesh was put back in place for a further 10 minutes to allow the resident to make social threats. Adolescent mice were exposed to social defeat on postnatal day (PND) 27, 30, 33 and 36, and adult mice on PND 47, 50, 53 and 56. The same protocol was used for the exploration groups but without a “resident” mouse in the cage. Following this last phase, the mice remained in the animal facility for three weeks, after which time the behavioral tests began. The second phase of each social defeat protocol was video-recorded and ethologically analyzed. Behaviors relating to threat and attack were scored in resident mice and behaviors relating to avoidance/flee and defensive/submissive were evaluated in intruder mice.

#### Conditioned place preference (CPP)

For place conditioning, eight identical Plexiglas boxes were employed with two equally-sized compartments (30.7 cm long × 31.5 cm wide × 34.5 cm high), which were divided by a gray central area (13.8 cm long × 31.5 cm wide × 34.5 cm high). The compartments had contrasting colored walls (black vs white) and different floor textures (fine grid for the black compartment and wide grid for the white one). The animals’ positions and their crossings from compartment to compartment were recorded by means of four infrared light beams in each compartment of the box and six in the central area. The equipment was controlled by three IBM PC computers using MONPRE 2Z software (CIBERTEC, S.A., Spain).

Place conditioning, which consisted of three phases, was carried out during the dark on 3 consecutive days. On day 3, the time spent in each compartment was recorded. This procedure was carried out during the dark cycle following a procedure that was unbiased in terms of initial spontaneous preference [77]. During the first phase—or preconditioning (Pre-C)—mice had access to both compartments of the apparatus for a period of 900 s per day. The animals that showed a strong unconditioned aversion (less than 33% of the session time; i.e. 250 s) or preference (over 67% of the session time; i.e. 650s) for any compartment were excluded for the remainder of the study. In each group, half of the animals received the drug or vehicle in one compartment and the other half in the other compartment. An ANOVA showed there was no significant difference between the time spent in the drug-associated and the vehicle-associated compartments during the Pre-C phase. In the second phase (conditioning, 4 days), animals were conditioned with either cocaine or saline. The mice were then administered an injection of physiological saline before being confined to the vehicle-associated compartment for 30 min. After 4 hours, the animals received cocaine immediately before an extra 30 minutes of confinement in the drug-associated compartment. The central area was rendered inaccessible by guillotine doors during conditioning. In the third phase—or postconditioning (Post-C)—which took place on day 8, the guillotine doors dividing the two compartments were lifted, and the time that the untreated mice spent in each compartment was recorded during a 900 s observation period. The difference in seconds between the time spent in the drug-associated compartment during the Post-C and Pre-C tests is a measure of the degree of conditioning induced by the drug. If this difference is positive, the drug has prompted a preference for the drug-associated compartment, while the opposite indicates an aversion.

#### Tissue sampling

On PND 63, mice were anesthetized with a mixture of medetomidine (Domtor, Esteve Veterinaria) and ketamine hydrochloride (Ketolar, Pfizer). They were then transcardially perfused with physiological saline 0.9% at 4°C followed by 4% paraformaldehyde (PFA, Merck) in phosphate buffer (PB, 0.1 M, pH7.2). The brains were removed and post-fixed in PFA 4% for 48h at 4°C and were then washed in PB (0.1M, pH 7.2). They were washed a further three times, for 30 min each time, in PB (0.1 M, pH 7.2), and cryoprotected in 11% sucrose in phosphate buffer saline (PBS, 0.1 M, pH 7.4) at 4°C for 48 h, after which they were transferred to PBS containing 33% sucrose and conserved at 4°C for 48 h. Finally, samples were frozen and kept at -30°C until further use.

For immunostaining techniques brains were frozen sectioned into coronal sections of 25 μm using a cryostat microtome (CM-3050, Leica, Germany). Tissue sections were collected in gelatin-coated slides (4 slices per slide), air dried and stored at -30°C.

#### Immunohistochemistry

For immunohistochemical detection separate sets of sections were serum blocked and incubated in rabbit antibodies to GFAP (1:2000), AbD (Dako, Ref: Z0334); Iba1 (1:100) (Wako Chemicals, Ref: 019–19741) and mouse monoclonal antibodies to NeuN (1:100) (Millipore Clone A60, Ref: MAB377) and Synaptophysin (1:800) (Sigma Clon SPV-38, Ref: S5768) for 24-48H at 4°C. After three washes (for 10 minutes each time), sections were incubated for 1h at room temperature with appropriate secondary antibodies: Goat anti-Rabbit biotinylated IgG (H+L)(1:200) (Thermofisher, Ref: NJ1613753) and Goat anti-Mouse Biotinylated IgG (H+L) (1:200) (Vector Laboratories Inc, Ref: BA9200).

Slides immunostained for GFAP, Iba1, NeuN, and Sinaptophysin were observed in a Zeiss Axio-Imager 2. Images where captured with the same adjustments of contrast and brightness with a Zeiss Axiocam Camera and processed using Axiovision 40 V 4.1 software (Carl Zeiss vision GMBH).

### Quantitative analysis

All quantification analyses of NeuN, GFAP, synaptophysin and Iba1^+^ cells were performed in the prelimbic cortex (PrL) and nucleus accumbens core (NAc), with high resolution digital micrographs captured under the 20x or 10x magnification objective. Neuroanatomical sites were identified with the Paxinos and Franklin mouse brain atlas [78], the anterior-posterior localization from Bregma of the analyzed areas was: PrL, 1,98–1,54 mm; NAc core, 1,42–0,74 mm. Slices count areas were calculated in mMC (426 x 0.338 mm for 20 magnification and 0.85 x 0.67 mm for 10x) and immunopositive cell count was expressed as number of cells/mMC for the quantification of the number of GFAP+ cells and each of the morphological types of Iba1^+^ cells. The densitometric studies were performed for the quantification of total Iba1^+^, NeuN and synaptophysin + cells, by measuring the optical density (OD) of the selected area with the software Image J (NIH, USA). We measured the total OD and subtracted the OD of the unmarked tissue, results were express as optical density (arbitrary units).

#### Quantification GFAP+

To investigate whether RSD affects astrocytes in mice, we analyzed the expression of glial fibrillary acidic protein (GFAP). This cytoskeletal protein is a general marker of mature astrocytes in the brain, except in neurogenic niches, where it is expressed by neural stem cells that are differentiated from mature astrocytes by the negative expression of S100β [79, 80]. Presence was determined by counting the number of immunoreactive GFAP cells in a whole area of the sections pictured at 20X magnification. Results are expressed as number of positive GFAP+/mm2.

#### Densitometry and morphology of Iba1^+^

Microglial response was evaluated by quantification of Iba1^+^ cells, a calcium-binding protein specifically expressed in microglia [81]. We also evaluated changes in microglial morphology, since the resting phenotype is characterized by a ramified morphology with a small round soma, but microglia changes to an amoeboid structure in response to harmful stimuli [55, 58]. Immunoreactive cells were densitometered in a selected area of the sections pictured at 20X magnification. Microglia morphology analysis was performed according to established morphological criteria. Cells were classified in five morphological types [82]: type I, cells with few cellular processes (two or less); type II, cells showing three to five processes; type III, cells with more than 5 processes and a small cell body; type IV, cells with large somas and retracted and thicker processes; and type V, cells with amoeboid cell body, numerous short processes and intense Iba1^+^ immunostaining. Iba1^+^-immunoreactive cells types III, IV and type V were counted together.

#### Densitometry of NeuN and synaptophysin

We identified neurons with the nuclear antigen NeuN and the synaptic protein synaptophysin. NeuN immunoreactivity is considered a neuronal marker, and weak NeuN immunostaining is associated with vulnerability and neuronal loss [83, 84]. Immunoreactive NeuN^+^ densitometry was recorded with micrographs taken with a 20X magnification objective, while anti-CB1 and anti- synaptophysin was performed with a 10X magnification objective.

### Statistical analyses

Statistical significance for immunoreactivity expression, for the morphotype Iba1^+^ and for the CPP data (difference of time spent in the drug-paired compartment in Post-C vs. Pre-C tests) was determined by a mixed two-way ANOVA with two between-subjects variables—Stress, with two levels (RSD and EXP), and cocaine treatment, with three levels (Saline, C1, C25). Prior to this, the Shapiro-Wilk normality test and Levene homoscedasticity test were performed to analyze the data obtained for immunoreactivity expression and the morphotype Iba1^+^, which were transformed to satisfy the normality assumption for the ANOVA. Post hoc comparisons were performed with Bonferroni tests. The value of the effect size was evaluated by the partial square ETA. The results are reported as mean ± S.E.M. All Statistical analyses were performed using the SPSS 19.0 software package (SPSS Inc., Chicago, IL, USA).

## Results

### Repeated social defeat in combination with cocaine preference reduces microglia and promotes long-term changes in inflammatory reactivity

**Fig 1:**
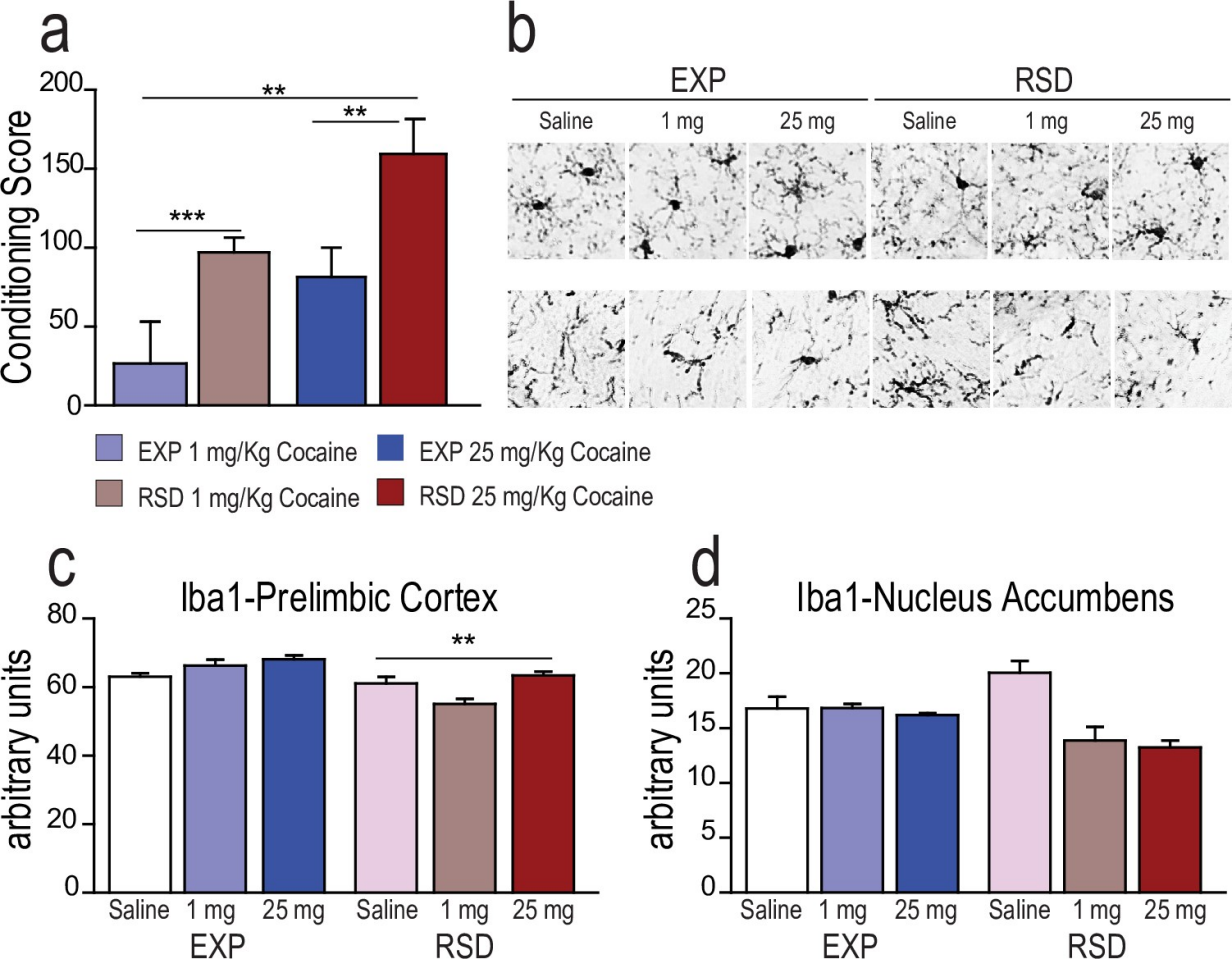
a) Effects of Repeated Social Defeat (RSD) during adolescence on 1 and 25 mg/kg of cocaine-induced CPP. *** p< 0.001, **p< 0.01; b) Effects of Repeated Social Defeat (RSD) during adolescence and cocaine treatment (1 and 25 mg/kg) on the expression of Iba1^+^ cells in the PrL c) and NAc d) in adult male mice. ** p<0.05 general stress effect.

The ANOVA of the effects of RSD on cocaine-induced CPP in adolescent mice (Fig 1A) showed an effect of the variables Stress (F1,50 = 12.086; p<0.001) and Cocaine dose (F1,50 = 7.228; p<0.01). Mice socially defeated during adolescence presented a higher conditioning score than their respective non-stressed controls (p< 0.001 for 1 mg/kg dose and p< 0.01 for 25 mg/kg) (effect size 0.195). As expected, mice conditioned with 25 mg/kg of cocaine gave higher conditioning scores than those conditioned with 1 mg/kg (p<0.01)(effect size 0.126). As we have previously reported (Rodriguez-Arias et al, 2017), only socially stressed mice became conditioned at very low doses, confirming an effect of stress induced-drug sensitization.

Next, we quantified the number of microglia and their degree of reactivity in two key structures (NAc and PrL cortex) that process the rewarding effects of cocaine: PrL and NAc. ANOVA of Iba1^+^ quantification in the PrL showed a significant effect of the variable Stress (F 2,24 = 6.298; p< 0.05), where Iba1^+^ expression was decreased in all socially defeated groups (Fig 1B, 1C and 1D).

**Fig 2:**
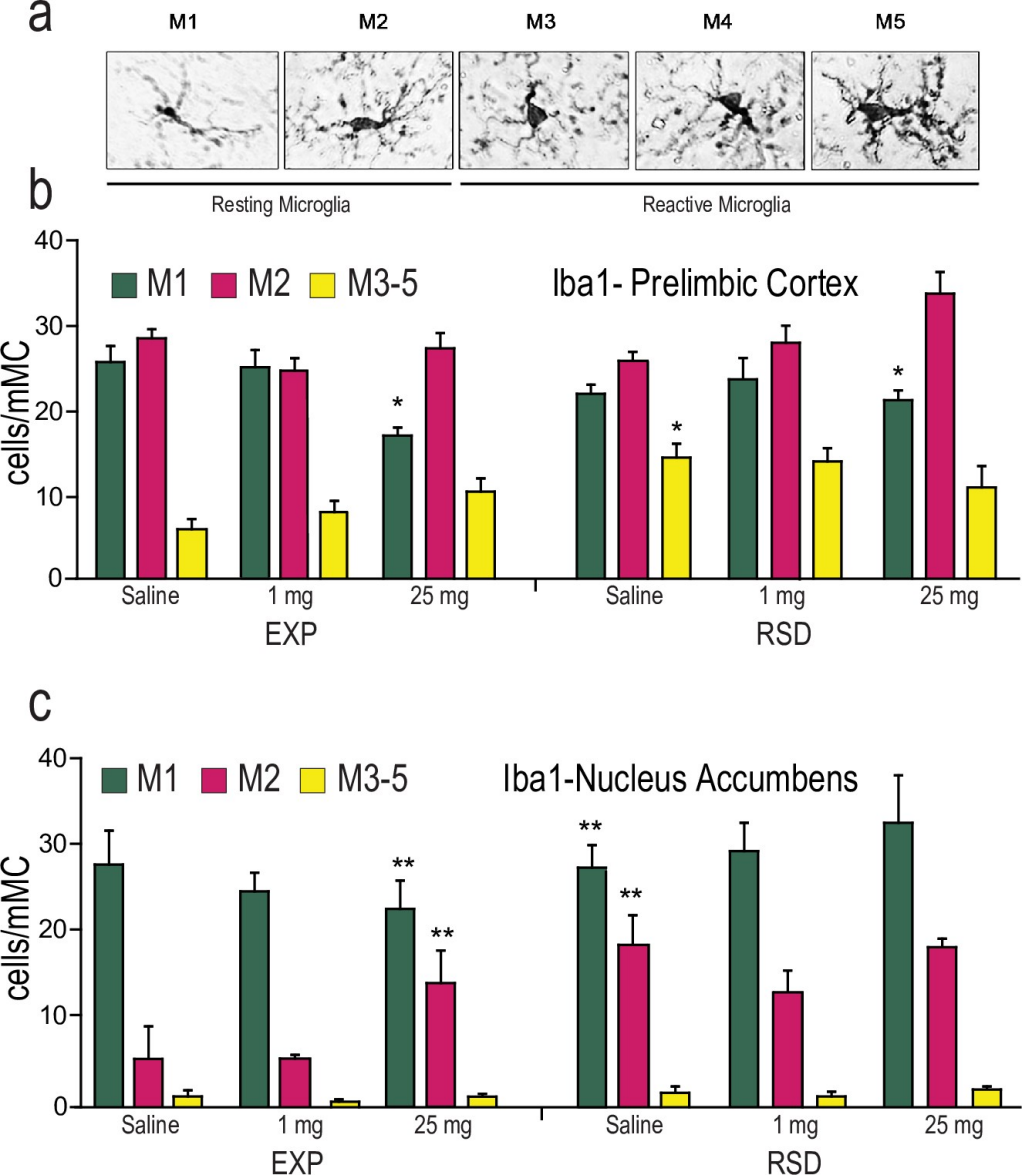
a) Effects of Repeated Social Defeat (RSD) during adolescence and cocaine treatment (1 and 25 mg/kg) on different microglia morphotypes based on activity in the PrL b) and NAc c) in adult male mice. * p<0.05, ** p< 0.001 with respect Exp Saline group.

Microglia phenotype was characterized in an inflammatory gradient scale from M1 (less inflammatory response) to M5 (more inflammatory response). M3-M5 were considered reactive microglia (Fig 2A). When this parameter was analyzed with ANOVA, a significant interaction was detected between Stress X Cocaine (F2,24 = 5.753; p<0.01) in PrL. RSD mice treated with saline displayed a larger number of Iba1^+^ cells of M3, M4 and M5 morphotypes than animals also treated with saline but which did not experience stress during adolescence (p< 0.05) (Fig 2B). In addition to this, the highest dose of cocaine produced an activation of microglia in all mice, even the non-stressed ones (decreased M1 morphotype p<0.05 in all cases).

ANOVA displayed a significant effect of the interaction between the variables Stress X Cocaine in the NAc (F2,24 = 3.621; p<0.05). Post-hoc analysis revealed a higher number of Iba1^+^ in M2 phase and a decrease of those in M1, in non-stressed animals treated with 25 mg/kg cocaine, showing once again the deleterious effect of this concentration of drug on microglia. Curiously, socially defeated animals treated with saline (p<0.01 in both cases) also showed a rise of M2 Iba1^+^ cells and a decrease in those in M1 (Fig 2C), with no changes in those in M3-5.

Considered together, these results show that social stress induced during adolescence has a long-term effect of a decrease in numbers and increased reactivity of microglia during adulthood. This is compatible with an inflammatory environment and heightened cellular vulnerability.

### Repeated social defeat in adolescence decreases the number of astrocytes in adult NAc and leaves neurons more vulnerable to eventual drug insults

**Fig 3:**
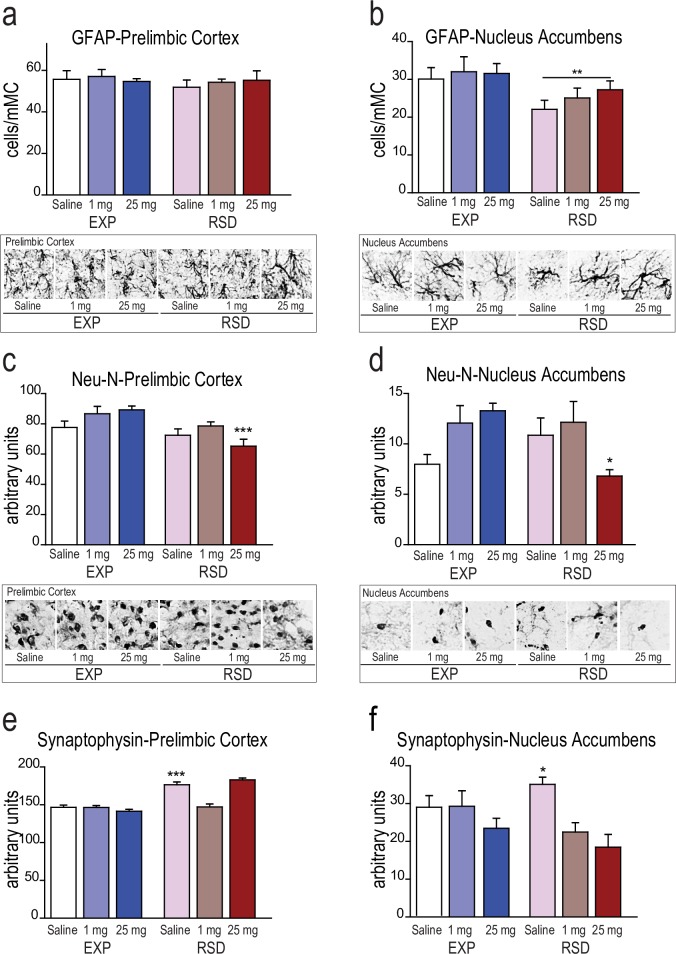
Effects of Repeated Social Defeat (RSD) during adolescence and cocaine treatment (1 and 25 mg/kg) on the number of GFAP+ a) and b); NeuN c) and d); and synaptophysin e) and f) cells in the PrL and NAc in adult male mice. * p<0.05 significant difference RSD cocaine treated groups; ** p> 0.01 general stress effect; *** p<0.001 significant difference vs their respective saline control group.

Quantification of GFAP^+^ astrocytes did not reveal any difference in the PrL cortex, but they were significantly reduced in the NAc (F1,24 = 9.22; p< 0.01). Repeated exposure to social stress reduced the number of GFAP^+^ cells in this structure in all treated groups compared with non-socially stressed animals (Fig 3A and 3B). Given that astrocytes are the main supporting glia for neurons, we sought to evaluate how neurons were affected by this observed undermining benefits of the glia.

Consequently, when we measured the number of neurons (NeuN^+^ cells) remaining in the PrL and NAc, we observed a significant downregulation with the highest cocaine dose in the stress conditions (Fig 3C and 3D). ANOVA revealed a significant effect of the interaction between the variables Stress X Cocaine in the PrL (F2,24 = 4.102; p<0.05) and NAc (F2,24 = 7.172; p<0.01). Post-hoc comparisons showed that animals experiencing RSD and treated with the highest dose of cocaine had lost more neurons than those receiving the same dose but not undergoing stress (p< 0.001 in PrL and p< 0.05 in NAc), suggesting a combined effect in which a high dose of cocaine produces an insult whose consequences are more pronounced when social stress conditions have previously been experienced.

Finally, we analyzed the functional reliability of the remaining neurons with the synaptic protein synaptophysin. Two-way ANOVA showed a significant effect of the interaction between the variables Stress X Cocaine in the PrL (F2,24 = 28.826; p<0.001) and NAc (F2,24 = 3.564; p<0.05). Synaptophysin expression in the PrL cortex was higher in defeated animals treated with saline (Fig 3E), indicating a stress-dependent effect on this synaptic protein (p< 0.001). Interestingly, socially defeated animals treated with cocaine exhibited lower synaptophysin levels in the NAc than RSD animals treated with saline (Fig 3F) suggesting a different sensitivity to experimental variables in this nucleus (p< 0.05).

These results indicate that supporting glia cells are highly affected by stress experienced in early periods of life, with their numbers being reduced and their capacity to support neurons undermined. The combination of raised inflammatory signals and microglia reactivity, in addition to a decrease in astrocyte protection, results in high neuronal vulnerability in stress- and drug-sensitive areas.

## Discussion

Repeated Social Defeat is recognized as a multisystem-impacting stressor. Most research in the field has been performed in animals socially defeated during adulthood and have evaluated effects during a short time after the last defeat. However, the long-lasting effects of RSD experienced during adolescence have not been well studied. Our study confirms that RSD during adolescence induces increases in the conditioned rewarding effects of cocaine in adulthood. Stressed mice developed CPP with a non-effective dose of cocaine (1 mg/kg) and showed significantly higher preference for an effective dose than their non-stressed counterparts.

As we have observed in previous experiments [22, 23], mice exposed repeatedly to social defeat during adolescence developed CPP with a dose of cocaine (1mg/kg) that was non-effective in control animals. On the other hand, 25mg/kg of cocaine induced CPP in all groups, though defeated mice showed a greater preference than control animals. When the conditioned rewarding effects of amphetamine were studied in adolescent-defeated animals by Burke and co-workers, similar results were obtained [85]. Social defeat during adolescence has also been demonstrated to induce an increase in the acquisition of intravenous cocaine and oral ethanol self-administration in adulthood [22, 29], and shows particularities when compared to that experienced in adult animals. We have previously shown that the effects of social defeat aggression are less intense in adolescent mice, which show a significant increase in corticosterone levels only after the fourth encounter, in contrast with the sharp rise observed in adult mice after the first encounter [23]. Our results indicate that RSD increases the sensitivity of mice to the conditioned rewarding effects of cocaine. Although mice defeated in adolescence showed attenuated amphetamine-induced increases of DA in the medial prefrontal cortex, there was an increased response to amphetamine in the NAc, thus supporting our hypothesis [85].

In the present study we show that the increase in the conditioned rewarding effects of cocaine is accompanied by alterations in glial and neuronal markers. We have evaluated two brain areas implicated in the rewarding effects of cocaine: the PrL cortex and the NAc. Under non-stress conditions, neither 1 nor 25 mg/kg of cocaine induced any effect per se on any of the parameters studied (glia and neuronal profiles); however, social defeat induced profound changes when combined with a dose of cocaine, and in the absence of it.

Defeated mice showed changes in microglia activation in both structures 3 weeks after the last social defeat; a decrease of Iba1^+^ cells in the PrL cortex was complemented by a decrease in morphotypes 1 and 2 and increases in morphotypes 3, 4 and 5 in both structures. Few studies have explored the effect of social stress on Iba1^+^ expression, and all have been performed in adult animals and immediately after the last social defeat. Said studies have revealed an increased expression of microglial marker Iba1^+^ in different brain areas, [86] and a heightened response after a secondary immune challenge [87]. The conclusions of prior research are not consistent regarding the effect of stress on microglial activation. Most studies have reported changes in microglial activation in response to stress, but there is not a consensus regarding the characteristics of said changes. For instance, stress has been reported to both de-ramify and hyper-ramify microglia, to increase and decrease cell size, and to increase and decrease Iba1^+^ levels [86, 87, 88, 89, 90].

Under our experimental conditions (long-lasting effects of adolescent stress), we found only a slight decrease of Iba1^+^ immunoreactive cells in the PrL, and no changes in the NAc. On the other hand, we observed an increase of the most active morphotypes of microglia in response to stress and cocaine treatment. Defeated animals exhibited a more pronounced activation of microglia, with a decrease of the morphotypes M1 and M2 and an increase of M3-5, all three being the most active forms. These results are in line with other evidence associating morphological changes in microglia with an inflammatory profile after social defeat [91]. To sum up, in our experimental conditions, the total number of Iba1^+^ microglial cells is reduced by RSD, and there is a decrease in resting phenotypes in both the PrL and NAc.

We also found astrocytes to be affected, since all defeated mice showed a decrease in GFAP^+^ cells in the NAc. Astrocytes express glucocorticoid receptors [92] and are responsive to stress [67], with corticosterone inducing a decrease in GFAP levels in the rat brain [93, 94]. Therefore, we believe that the decreased number of astrocytes after RSD is a sign of long-term cell damage. In agreement with our results, a previous report by Araya-Callís and co-workers [95] showed that, after daily social defeat experienced over a 5-week period, there was a decrease in GFAP expression in the hippocampus 24h after the last defeat. However, other types of stress, like activity stress, chronic variable stress or thermal stress, have been shown to increase GFAP immunoreactivity in several brain regions, which may indicate stimulation of reactive astrocytes [96, 97, 98]. Hence, social stress seems to exert a different effect on astrocyte function. In line with this, a decrease in GFAP+ astrocytes is observed in the medial prefrontal cortex after juvenile separation [99], in the hippocampus after chronic social defeat [100], and in the neocortex after chronic mild stress [101].

Finally, we observed that neuronal synaptic function was also modified by RSD. The neuronal marker NeuN was diminished in defeated mice after administration of the highest cocaine dose. Previous research has described how chronic social stress promotes a decrease in neuronal populations—assessed by the NeuN marker—in several brain structures, including the hippocampus [102, 103]. Once again, the aforementioned results were obtained immediately after social defeat in adult animals. In the present study, we demonstrate that this effect is also present in adolescent animals, is prolonged in time, and is potentiated by concomitant cocaine administration.

In our experiments, the changes in synaptophysin depended on the structure studied. While an increase was observed in the PrL of defeated mice, a decrease was detected in the NAc of those treated with cocaine. Sometimes synaptophysin signal in the cortex is not easily associated with a specific nucleus, and several variables can affect its quantification. Neurons in both areas may show different degrees of vulnerability to damage, in which case the synaptophysin signal would be diminished by a strong neuronal insult, while the projection of neurons from other structures to the cortex could be more resilient to experimental variables, in which case quantification would reveal a mix of resident and projecting neurons. In addition to this, social defeat induces abnormal structural plasticity of dendrites and spines in different brain structures [104]. For example, adult animals exposed for 5 weeks to chronic unpredictable stress exhibit a short-term decrease in synaptophysin density in the hippocampus and prefrontal cortex [105]. Conversely, in our stress protocol, there was a trend for synaptophysin density to increase, though it was significant only in the PrL cortex. In line with our results, the hippocampal expression of synaptophysin was found not to differ in adult rats exposed to social instability stress during adolescence [106]. However, isolation during adolescence was reported to induce a reduction in synaptophysin in the infralimbic cortex and cingulate gyrus in adulthood [107]. We also found that cocaine treatment had no significant effect on synaptophysin expression in the NAc of control animals, but did induce a significant decrease in socially stressed animals. In accordance with these results, other authors failed to observe changes in synaptophysin expression in the NAc after five-day treatment with a dose of 10mg/kg [108].

Finally, we have to consider the limitations of the model employed. Although men are more deeply affected by social stress at a physiological level [109], women show higher rates of anxiety and fear. Since most female rodents do not express spontaneous aggression, one of the major disadvantages of the social defeat paradigm is that it is principally designed for male rodents. Furthermore, we have to take into consideration that the SD model emphasizes the social aspect of stress, but it produces both physical and social stress. The physical stress suffered by the animal also has a bearing, and it is difficult to separate the two types of stress within this model [110].

Considered as a whole these results suggest that social stress experience modulates vulnerability to suffer a loss of glia-supporting cells and functional neuronal synaptic density due to drug consumption in later life.

## Supporting information

S1 File Experimental data. (XLSX)
